# Physicochemical Characteristics of Phospholipid Vesicles for Spirulina-Based Dietary Supplement Delivery

**DOI:** 10.3390/molecules30122581

**Published:** 2025-06-13

**Authors:** Massimo Milia, Ines Castangia, Francesco Corrias, Matteo Aroffu, Mattia Casula, Maria Letizia Manca, Maria Manconi, Hamza Bouakline, Alberto Angioni

**Affiliations:** 1 Department of Life and Environmental Science, University Campus of Monserrato, University of Cagliari, SS 554, 09042 Cagliari, Italy; max_milia@hotmail.it (M.M.); francesco.corrias@unica.it (F.C.); matteo.aroffu@unica.it (M.A.); mattiacasula92@gmail.com (M.C.); marial.manca@unica.it (M.L.M.); maria.manconi@unica.it (M.M.); 2 Laboratory of Applied Chemistry and Environment, Faculty of Sciences, University Mohamed First, Oujda 60000, Morocco; h.bouakline@ump.ac.ma

**Keywords:** spirulina extract, physicochemical properties, liposomes, bilosomes, gelatin-enriched bilosomes

## Abstract

Spirulina (*Arthrospira platensis*) is a microalga widely used as a dietary supplement in sports nutrition and in treating metabolic diseases such as diabetes, obesity and metabolic syndrome. Spirulina’s cell structure limits digestibility and reduces the availability of bioactive compounds. The extraction processes, coupled with encapsulation, can enhance the bioavailability of nutritional and antioxidant compounds, protecting them from degradation, preserving their functional activity, and supporting controlled release. The physicochemical properties of liposomes (Lps), bilosomes (Bls), and gelatin-enriched bilosomes (G-Bls) with incorporated Spirulina extracts were investigated. The delivery systems exhibited small particle size (101.8 ± 0.5 to 129.7 ± 1.2 nm), homogeneous distribution (polydispersity index (PDI) 0.17 ± 6.67 to 0.33 ± 9.06), negative surface charges (−31.9 ± 5.2 to 31.1 ± 6.4 mV), and high entrapment efficiency (>80%). G-Bls demonstrated effective retention of the extract, with a low release rate at pH 1.2 (41.8% ± 6.1) and controlled release at pH 7.0 (52.5% ± 3.0). Biocompatibility studies on Caco-2 cells showed that G-Bls maintained high cell viability at 200 μg·mL^−1^ (87.89% ± 10.35) and significantly mitigated H_2_O_2_-induced oxidative stress at 20 and 200 μg·mL^−1^, increasing cell viability by 23.47% and 19.28%. G-Bls are a promising delivery system for enhancing the stability, bioavailability, and protective effects of Spirulina extracts, supporting their potential application in dietary supplements aimed at promoting sports performance and recovery, mitigating exercise-induced oxidative stress, and managing metabolic disorders.

## 1. Introduction

Arthrospira platensis (Spirulina) is a filamentous, spiral-shaped prokaryotic cyanobacterium capable of nitrogen fixation and thriving in extreme environments, withstanding elevated temperatures, light irradiation, and strong alkaline conditions. Spirulina is recognized for its high nutritional value characterized by a high protein content (50–70% DW), carbohydrates (15–20% DW), and important bioactive compounds including fatty acids, carotenoids, phycocyanin, and polyphenols [1,2,3]. These bioactive compounds have significant antioxidant and anti-inflammatory properties, contributing to overall human health and well-being. Extensive research has explored the potential of Spirulina in mitigating metabolic disorders such as hyperlipidemia, atherosclerosis, hypertension, diabetes, and weight control [4,5,6,7,8,9,10]. It is recognized as a useful sports supplement aiding performance, especially during intense physical activity, and physical recovery [11,12,13]. In addition, Spirulina showed a beneficial impact on gut microbiota with promising uses in the prevention and treatment of chronic diseases, optimizing physiological and metabolic outcomes when dietary and lifestyle modifications are applied [14,15,16,17,18]. The oral bioavailability of active compounds represents the bottleneck for Spirulina consumption due to the rigid peptidoglycan cell wall combined with protein–pigment complexes. Moreover, Spirulina bioactive compounds can be degraded by enzymatic activity within the gastrointestinal tract and their limited intestinal permeability. Therefore, the development of innovative extractive techniques, designed to increase its nutritional potential, and appropriate dietary supplements able to protect Spirulina bioactive compounds and boost their bioavailability represent critical challenges [19,20,21,22,23].

Dietary supplements are regulated in the European Union by the General Food Law Regulation (EC) No 178/2002 and are defined as concentrated sources of nutrients and bioactive compounds, such as vitamins, minerals, amino acids, essential fatty acids, and plant extracts [24]. Dietary supplements can prevent physiological and nutritional deficiencies and are classified according to type and function. The most important factors that affect nutrient intake are ensuring proper nutrition, reducing the risk of age-related disorders, and protecting body tissues. [25]. Primary consumers include active adults, particularly women (25–65 years), from medium-high-level school education, people affected by metabolic conditions, and young individuals with healthy lifestyles [26,27].

Among the various drug delivery systems, liposomes are the most studied. Liposomes are small, spherical vesicles with one or more phospholipid bilayers that mimic cell membranes, composed of cholesterol and biocompatible phospholipids, with an average diameter below 1 μm. Liposomes have hydrophilic and lipophilic properties and are extensively used to encapsulate proteins, carbohydrates, lipids, vitamins, and minerals, facilitating targeted delivery, sustained release, and protection against chemical degradation, thereby enhancing metabolic efficacy [28,29]. There are many different types of liposomes and many preparatory methods, which mostly include lipid solubilization by organic solvents, forming a thin lipid film by the evaporation, hydration and physicochemical characterization of liposome properties [29]. Conventional liposomes have demonstrated instability within the gastrointestinal tract and diminished efficiency in retaining active agents, which limits their therapeutic application [30]. To address these limitations, advanced lipid vesicles such as bile-salt enriched liposomes (Bls) have been developed. These vesicles enhanced flexibility and structural integrity, achieving higher entrapment efficiency, particularly for poorly water-soluble compounds [31]. The strong structures of these vesicles minimize premature leakage under harsh acidic and enzymatic gastrointestinal conditions and improve permeation across the intestinal mucosa, enhancing oral bioavailability [32,33,34]. In recent years, increasing attention has been given to developing gelatin-based phospholipid vesicles. Gelatin is a natural biopolymer derived from collagen hydrolysis. It is highly valued for its biocompatibility, biodegradability, and low immunogenicity and is classified as “Generally Recognized as Safe” (GRAS) by the U.S. FDA. Its main structure comprises a three-dimensional shape formed for 50% by three amino acids (glycine, proline, hydroxyproline) and lower amounts of alanine, arginine, and aspartic and glutamic acid. Among various biopolymers, gelatin stands out for its ability to maintain particle size distribution and entrapment efficiency. Gelatin-based delivery systems enhance oral bioavailability and facilitate the uptake of active pharmaceutical ingredients by protecting them during gastrointestinal transit [35,36].

However, aggregation and sedimentation in aqueous dispersions and potential oxidation and lipid degradation persist, requiring further stabilization strategies [37]. Freeze-drying techniques utilizing sugars (e.g., mannitol, trehalose, or sucrose) or biopolymers (chitosan or alginate) as cryoprotectants have demonstrated significant efficacy in maintaining particle size distribution and entrapment efficiency and reducing aggregation and degradation, thereby enhancing the long-term stability of the formulations [38].

In this study, different delivery systems consisting of liposomes (Lps), bilosomes (Bls), and gelatin-enriched bilosomes (G-Bls) incorporating Spirulina extract were developed to enhance the stability, oral bioavailability, and controlled release of the bioactive compounds. The phospholipid vesicles were combined with mannitol as a cryoprotectant and freeze-dried to improve storage stability and shelf life.

The physicochemical properties of the phospholipidic vesicles were evaluated in terms of mean diameter (MD), the polydispersity index (PDI), surface charge (ζ potential), entrapment efficiency (EE%), shelf life, release profile, biocompatibility, and cytoprotective effects against hydrogen peroxide-induced oxidative stress in Caco-2 cells.

## 2. Results and Discussion

Phospholipid vesicles for the oral delivery of biomacromolecules have the functions of providing protection from the intestinal environmental characteristics and carrying them through the gastrointestinal membrane.

In this paper, the performances of Lp, Bl and G-Bl vesicles incorporated with Spirulina extracts represent a promising delivery strategy for improving oral drug retention and controlled release in the small intestine, the intestinal lymphatic, and the colon [39].

Pre-formulation studies were carried out with different concentrations of the extract (5, 10, 20, 30, and 40 mg·mL^−1^); different amounts of Lipoid S75 (60 and 120 mg·mL^−1^), SDC (10 and 20 mg·mL^−1^), and gelatin (40, 80, and 120 mg·mL^−1^); and cryoprotectants (mannitol, sucrose, trehalose, lactose, and mannose). The optimal composition was selected based on vesicle stability, homogeneity, and entrapment performance and was used in the present study (data not reported).

Phospholipid vesicles loaded with Spirulina extract demonstrated favorable size between 101.8 and 129.7 nm, with PDIs of 0.17 and 0.33, indicating uniform size distribution [40], comparable negative surface charge (*p* > 0.05), colloidal stability against aggregation (ζ between −31.1 and −31.9 mV) [41], and an EE% of over 82.1% (Table 1).

The analysis showed no statistically significant differences (*p* > 0.05) in MD and the PDI between Lps and Bls, indicating a comparable size distribution. In contrast, G-Bls exhibited a significantly larger MD (129.7 ± 1.2 nm, *p* < 0.0001) and a higher PDI value (0.33, *p* < 0.01) due to the physicochemical properties of gelatin. These results align with the literature for Lps and Bls, accounting for 50–150 nm and up to 200 nm, respectively; moreover, PDI ≤ 0.3 indicates a uniform size distribution, and ζ potential above ± 30 mV suggests good stability against aggregation [41,42,43]. However, studies on Lps loaded with Spirulina extracts showed an almost double MD, ranging from 226.5 to 292; a higher PDI (0.38–0.52); and comparable ζ [44,45]. Slight variations in the physicochemical parameters could be attributable to different amounts of basic ingredients, types, and loaded extracts.

Determining the entrapment efficiency (EE%) of a drug is a challenging analytical task. Unlike the study of EE% of a single active ingredient, the total amount of drug encapsulated is a complicated issue when dealing with natural extracts such as plant or micro- and macroalgae extracts. HPLC analysis is a powerful technique used to qualify and quantify active ingredients in complex matrices. However, the considerable number of compounds in the Spirulina extract and the different chemical classes limit the performance of HPLC. Therefore, the entrapment efficiency (EE%) was assessed using the DPPH assay, which provides an indirect functional estimate of the bioactive compound content retained within the vesicles. To further substantiate these findings, the EE% was confirmed by quantifying total polyphenols, chlorophyll a (Chl a), and total carotenoids. No statistically significant differences were observed among the tested formulations (*p* > 0.05), with EE% values ranging from 82.1 ± 2.7% for G-Bls (Tot polyphenols) to 99.2 ± 11.5% for Lps (Tot carot), indicating a consistently high encapsulation performance across different bioactive components [46]. Multiple studies have assessed the high entrapment efficiency of Spirulina bioactive compounds in nanocarrier systems. Machado et al. and Păvăloiu et al. reported over 80% EE% of polyphenols in Lps, highlighting their strong interaction with the liposomal bilayer [44,47]. Comparable results have been reported for Chl a in protein-based particles (83.6–96.3%) and β-carotene (99% in Lps and 85.2–89.5% in Bls) [48,49].

**Figure 1:**
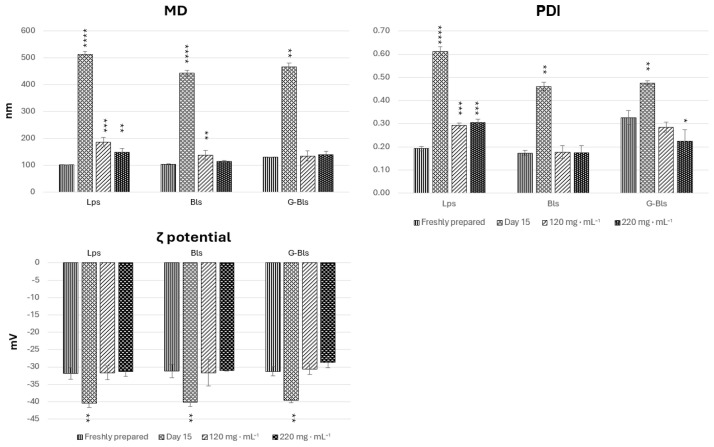
The physicochemical properties of the vesicles freshly prepared after 15 days of storage at 5 °C and with mannitol addition, followed by freeze-drying and rehydration. Data reported as mean values ±  standard deviations (n = 6). * *p* < 0.05, ** *p* < 0.01, *** *p* <0.001, and **** *p* < 0.0001 express the statistical difference between extract free dispersion (control) and other groups based on an ANOVA Tuckey comparison.

Following a 15-day storage period at 5 °C, all phospholipid vesicles exhibited significant physicochemical changes indicative of instability, in comparison with freshly prepared samples. MD increased markedly (*p* < 0.0001), reaching 512.3 ± 11.1 nm for Lps, 443.8 ± 10.5 nm for Bls, and 466.1 ± 13.9 nm for G-Bls, suggestive of aggregation. The PDI demonstrated a significant increase for Lps and Bls (*p* < 0.0001) and a more modest increase for G-Bls (*p* < 0.01), further reflecting colloidal destabilization. All formulations showed significant increases in negative ζ-potential (*p* < 0.01). Therefore, vesicle preparations were integrated with mannitol at 120 and 220 mg·mL^−1^. MD, PDI, and ζ potential were assessed immediately after freeze-drying for 72 h and rehydration in water (1:100 *v/v*) (Figure 1).

Mannitol influenced the physicochemical properties of the vesicles in a formulation-dependent manner. Lps and Bls showed increases in MD with respect to the fresh preparation with both concentrations of mannitol, even if the increase was higher in Lps, and in Bls there was no statistical differences between MD and the two concentrations of mannitol (*p* > 0.05). G-Bls showed a modest increase from 129.7 ± 1.5 nm to 134.7 ± 19.2 nm and 131.8 ± 12.75 nm without significant differences between the three experiments (*p* > 0.05). The PDI of Lps increased from 0.19 to 0.29 and 0.31 (*p* < 0.0001) upon increasing the mannitol concentration, indicating a high degree of size heterogeneity within the vesicle population. However, no statistically significant differences in mean particle size were observed among the mannitol-containing formulations. In contrast, Bls maintained a consistent PDI around 0.17–0.18 (*p* > 0.05), and G-Bls demonstrated a decreasing trend from 0.33 to 0.29 (*p* > 0.05) and 0.22 (*p* < 0.05), indicating improved uniformity with increasing mannitol content. The zeta potential remained stable across all phospholipid vesicles and within the two mannitol concentrations (*p* > 0.05) (Figure 1) (Table 2).

This trend suggests that mannitol promotes particle enlargement but enhances stabilization, ensures spatial isolation of particles, and prevents aggregation.

Freeze-dried solid samples were kept at 25 °C in the dark for 90 days to assess long-term stability. Afterwards, the vesicles were rehydrated with water and equilibrated at room temperature for 30 min. MD, the PDI, and the ζ potential were compared to the aqueous dispersions analyzed immediately after the freeze-drying process, and no significant differences were detected (*p* > 0.05). The addition of mannitol resulted in a pronounced stability enhancement, maintaining the desired physicochemical characteristics over extended storage [50]. This effect was more evident in G-Bls, which demonstrated optimal storability with negligible particle size variation, stable PDI, and minimal ζ potential changes, with gelatin preventing ice-crystal damage and preserving particle size [51,52]. Bls displayed intermediate stability, with sodium deoxycholate significantly enhancing freeze-drying stability by reducing interfacial tension and forming a protective lyophilic matrix [35], while Lps appeared the least stable due to the significant size increase (Table 2).

Gastrointestinal pH changes affected the structural stability of Lps and Bls, with them undergoing partial destabilization with respect to freshly prepared vesicles. In this study, simulated gastro and intestinal fluids were used as a simplified in vitro model to investigate the preliminary behavior of phospholipid vesicles in an aqueous environment mimicking the pH conditions of the gastrointestinal tract according to a previous work on similar systems [53]. Lps showed a significant increase in size and in the PDI after 2 h at pH 1.2 and after 6 h at pH 7.0 (*p* < 0.001) (Table 3).

Bls exhibited a significant increase in size at pH 1.2 (*p* < 0.001), whereas they decreased at pH 7.0 (*p* < 0.05), but the PDI increased at both pH. G-Bls showed the most substantial increase in size up to 239.7 ± 2.9 nm at pH 1.2 (*p* < 0.0001) and 221.2 ± 1.3 nm at pH 7.0; on the contrary, the PDI decreased to 0.22 at pH 1.2 (*p* < 0.01) and to 0.20 at pH 7.0 (*p* < 0.001, *p* > 0.05 compared to pH 1.2), suggesting that gelatin promoted significant swelling and size increase but ensured a more monodispersed population at the physiological pH.

**Figure 2:**
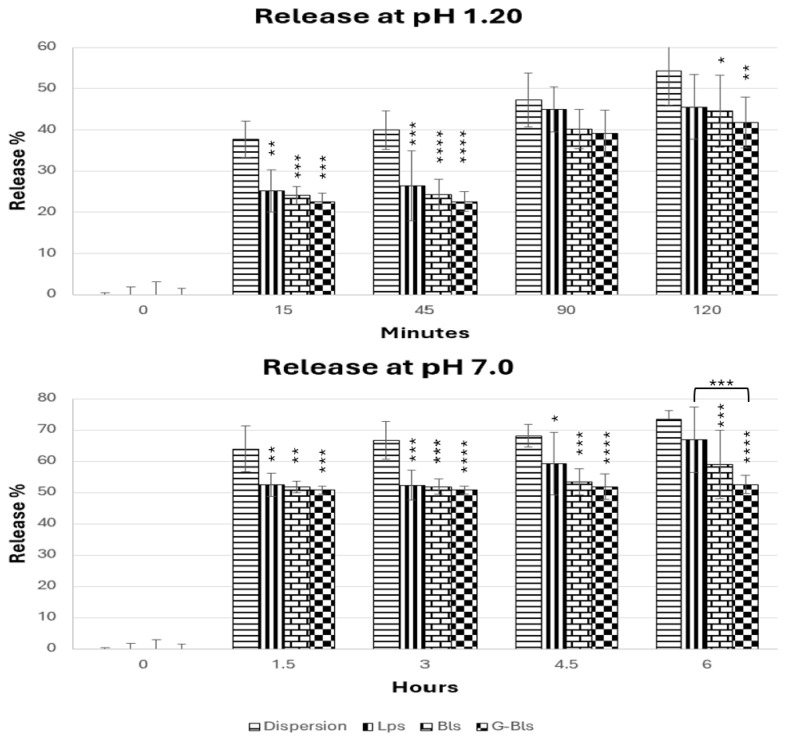
The release of the Spirulina extract from the dispersion and the vesicles at pH 1.20 for 2 h and at pH 7.00 for 6 h. * *p* < 0.05, ** *p* < 0.01, *** *p* <0.001, and **** *p* < 0.0001 express the statistical difference between extract free dispersion (control) and other groups based on the ANOVA Tuckey comparison.

At pH 1.2, the release followed the series Lps > Bls > G-Bls and was lower than the free dispersion (after 15 min ~24% vs. ~40%) (Figure 2). After 2 h, the extract dispersion released 54.2% ± 8.3, while vesicles showed similar release profiles, ranging from 41.8% ± 6.2 in G-Bls to 45.5% ± 8.7 (mean ± SD%) in Lps (*p* > 0.05), due to protonation-induced charge neutralization, highlighting the protective role of vesicles in limiting early drug exposure to the harsh gastric environment [54]. At pH 7.0, the releasing trend remained the same, with Lps being less efficient in preserving the extract. After 1.5 h, almost 50% was released from the vesicles, reaching 66.9 ± 15.6 after 6 h in Lps. The free dispersion reached 73.3% ± 2.9 at 6 h, whereas Bls (58.9% ± 10.9) and G-Bls showed a lower release, accounting for 52.5% ± 3.0 (mean ± SD%), suggesting that gelatin reinforced the vesicle structure, delaying diffusion. These trends are in line with MD and PDI studies. G-Bls were larger but more homogeneous and exhibited greater stability at low pHs but swelled at neutral pHs, forming a denser matrix that restricted rapid payload diffusion, potentially extending the therapeutic window and reducing dosing frequency [55,56].

**Figure 3:**
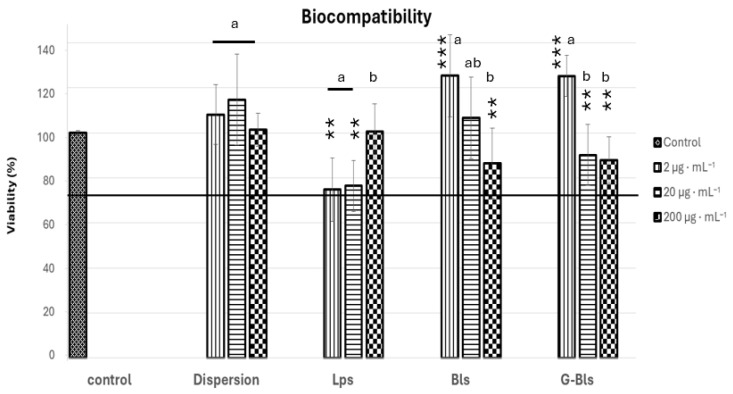
The viability of Caco-2 cells after 48 h of incubation with Spirulina extract dispersion or vesicles at three concentrations (2, 20, and 200 µg·mL^−1^). Data reported as mean values ± standard deviations (n = 6). ** *p* < 0.01, and *** *p* < 0.001 express the statistical difference between groups based on the ANOVA Tuckey comparison. Letters indicate statistical differences within each group based on ANOVA with Tukey’s post hoc test.

In vitro biocompatibility was assessed using human colon adenocarcinoma (Caco-2) cells at concentrations of Spirulina extract of 2, 20, and 200 μg·mL^−1^. Cell viability was determined using the MTT colorimetric test. The untreated control maintained a baseline viability of 100%, and similar results were achieved with the Spirulina extract dispersion, which showed a high biocompatibility at all tested concentrations, with viability ranging from 101.4% ± 7.4 (mean% ± SD%) to 114.8% ± 19.8 (*p* > 0.05 compared to control and within concentrations), confirming its safety and absence of toxic effects. Bls and G-Bls showed a similar trend with high biocompatibility at 2 μg·mL^−1^ (~125%), decreasing at higher concentrations of the extracts up to ~ 87%. Lps exhibited an inverse trend with lower viability at 2 μg·mL^−1^ and 20 μg·mL^−1^, around 75%, and an increased viability at 200 μg·mL^−1^, accounting for 100.5% ± 12.5 (Figure 3). According to EN ISO 10993-5:2009, cell viability values > 70% relative to the untreated control indicate good biocompatibility and the absence of cytotoxic effects [57]. All tested formulations exceeded this threshold, confirming their safety and overall compatibility with the Caco-2 cell line [58].

**Figure 4:**
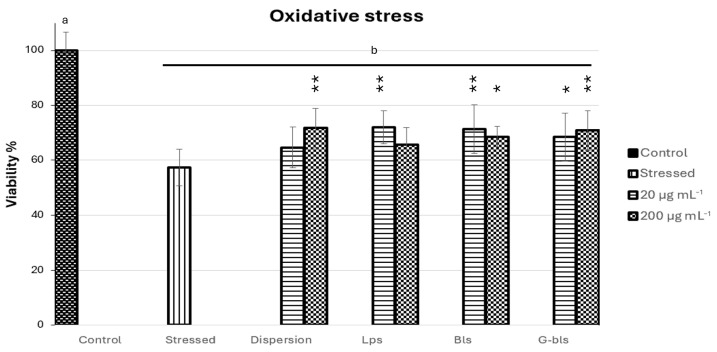
The viability of Caco-2 cells stressed with H_2_O_2_ and treated with Spirulina extract dispersion or vesicles at 20 and 200 μg·mL^−1^. Data are reported as mean percentages relative to untreated healthy cells (n = 8). Letters indicated statistical differences within untreated cells (control) and each group. * indicate statistical differences between H_2_O_2_ damaged cells (stressed) and cells treated with dispersion, Lps, Bls, and G-Bls (* *p* < 0.05, ** *p* < 0.01) based on ANOVA with Tukey’s post hoc test (*p* ˂ 0.05).

The protective effects of the Spirulina extract dispersion and its phospholipid vesicles (Lps, Bls, and G-Bls) were assessed against H_2_O_2_-induced oxidative stress in Caco-2 cells (Figure 4).

The stressed control group exhibited a significant decrease in cell viability to 57.3% ± 11.4 compared to untreated cells (*p* < 0.0001), confirming the cytotoxic impact of oxidative stress. Treatment with Spirulina extract dispersion at 20 μg·mL^−1^ did not significantly improve viability, although a slight increase was observed (64.6% ± 11.5; *p* > 0.05), while at 200 μg·mL^−1^ viability significantly increased by 25.0% (71.7% ± 10.1; *p* < 0.01), indicating a dose-dependent protective response. Lps showed a viability slightly improved by 14.5% (65.6% ± 9.5) at 20 μg·mL^−1^ with respect to stressed cells (*p* > 0.05) and by 25.4% (72.4% ± 9.1) at 200 μg·mL^−1^ (*p* < 0.01), suggesting enhanced delivery capacity at higher concentrations. Bls induced statistically significant improvements at both concentrations, with viability increasing by 19.52% (68.5% ± 5.5) at 20 μg·mL^−1^ (*p* < 0.05) and by 24.5% (71.4% ± 12.4) at 200 μg·mL^1^ (*p* < 0.01), reflecting efficient bioactive protection even at the lower dose.

G-Bls exhibited an inverse trend, with the highest protection observed at 200 μg·mL^−1^ (70.8% ± 10.2, *p* < 0.01 respect stressed cells) compared to 20 μg·mL^−1^ (68.4% ± 12.8, *p* < 0.05), corresponding to increases in viability of 23.5% and 19.3%, respectively, highlighting the modulatory role of gelatin in vesicle performance.

Overall, both free and incorporated Spirulina extract treatments conferred significant cytoprotection against oxidative damage. While no statistically significant differences were found between formulations, the data suggest that phospholipid vesicles preserved the antioxidant potential of the bioactive compounds, supporting their application as delivery systems in oxidative stress-related conditions.

## 3. Materials and Methods

### 3.1. Standards and Reagents

Methanol (MeOH), hydrochloric acid (HCl), and sodium hydroxide (NaOH) were ultra-residue solvents of analytical grade sodium deoxycholate (SDC), gelatine, cholesterol, NaCl, Na_2_HPO_4_, NaH_2_PO_4_, H_2_O_2_ (at 30%), and dimethyl sulfoxide (DMSO) purchased from Merck (Darmstadt, Germany). Lipoid S75 (soybean phospholipids with 70% phosphatidylcholine) was purchased from Lipoid GmbH (Ludwigshafen, Germany). Cell medium, fetal bovine serum, penicillin, streptomycin, fungizone, and MTT (3-(4,5-Dimethylthiazol-2-yl)-2,5-Diphenyltetrazolium Bromide) were purchased from Thermo-Fisher Scientific Inc. (Waltham, MA, USA). Double-deionized water, with a conductivity of less than 18.2 MΩ, was produced using a Milli-Q system (Millipore, Bedford, MA, USA). The simulated gastric fluid solution at pH 1.20 was prepared by dissolving 1.75 g of NaCl in 94 mL of Milli-Q water and adding 6 mL of HCl 1 M. The simulated intestinal fluid solution at pH 7.00 was prepared by dissolving 7.26 g of Na_2_HPO_4_, 3.56 g of NaH_2_PO_4_, and 17.54 g of NaCl in 1 L of Milli-Q water [57]. The phosphate-buffered saline solution (PBS) was prepared in the laboratory for final concentrations of 8 mM of Na_2_HPO_4_, 3 mM of NaH_2_PO_4_ 3 mM, and 154 mM of NaCl and pH 7.4. The Spirulina extract was obtained from dried matrix kindly supplied by the producer (LiveGreen, Società Agricola SRL, Arborea, Oristano, Sardinia, Italy). After mixing 1 g of Spirulina with 14 mL of PBS, the suspension was subjected to ultrasonic extraction (UAE) according to Milia et al. [59].

### 3.2. Phospholipid Vesicles

#### 3.2.1. Preparation

Phospholipid vesicles were prepared by mixing Lipoid S75 (120 mg·mL^−1^) and cholesterol (20 mg·mL^−1^) in a glass vial (Lps), adding 20 mg·mL^−1^ of SDC for Bls and a further 80 mg·mL^−1^ of gelatin for G-Bls.

The lipid mixtures were hydrated with 2 mL of PBS for 2 h to facilitate phospholipid swelling, followed by sonication (4 s on/2 s off, 15 cycles, 14 µm probe amplitude) using a high-intensity ultrasonic disintegrator (Soniprep 150, MSE Crowley, London, UK) to produce small and homogeneous vesicles. Spirulina extract (20 mg·mL^−1^) was subsequently incorporated into the pre-formed vesicles and subjected to a second sonication step (4 s on/2 s off, 7 cycles, 10 µm probe amplitude) and a 5 min rest interval between cycles (Table 4).

#### 3.2.2. Physical Characterization

Phospholipid vesicles’ mean diameters (MDs, nm), polydispersity indexes (PDIs), and surface charges were determined by photon correlation spectroscopy (Zetasizer ultra; Malvern Instruments, Worcestershire, UK). The surface charge (ζ potential, mV) was measured according to the mixed-mode measurement-phase analysis (M3-PALS) method. To ensure optical clarity and to prevent the particle attenuation of the laser beam, each sample was diluted with water (1:100) before analysis according to Castangia et al. [60].

To remove the unloaded extract, vesicle dispersions (1 mL) were dialyzed for 2 h in 1 L of distilled water at 25 °C (Spectra/Por^®^ dialysis tubes, 12–14 kDa MW cut-off, 3 nm pore size; Spectrum Laboratories Inc., Breda, The Netherlands). The entrapment efficiency (EE%) was determined by comparing the antioxidant activity before and after dialysis using the DPPH (2,2-diphenyl-1-picrylhydrazyl) colorimetric test according to Paseban et al. [61] and was further confirmed by quantifying chlorophyll a (Chl a), total carotenoids (total Carot), and total polyphenols.

#### 3.2.3. Long-Term Stability

Lps, Bls, and G-Bls vesicles were prepared using mannitol as a cryoprotectant at 120 and 220 mg·mL^−1^ (maximum solubility in water at 25 °C). The phospholipid vesicles were freeze-dried in an Operon freeze-dryer (FDB-8603, Operon Advantech, Suwon, Republic of Korea) at 0.4 mbar for 48 h and stored at room temperature (25 °C) in hermetically sealed dark glass vials for 90 days. The powder was rehydrated to its original volume (2 mL), and the mean vesicle diameter, polydispersity index, and surface charge were measured.

#### 3.2.4. PH Sensitivity of Phospholipid Vesicles

To assess the pH-dependent stability of the vesicles, Lps, Bls, and G-Bls dispersions were incubated at 1% in acidic medium simulating pH gastric conditions (pH 1.2) for 2 h and in neutral medium simulating pH intestinal conditions (pH 7.0) for 6 h at 37 °C. The incubation was performed in an orbital shaker–incubator (BS-1 ES20, BIOSAN, Rome, Italy) under constant stirring (170 rpm). After incubation, the MD and PDI were measured to assess vesicle stability under different pH conditions.

### 3.3. Biochemical Analysis

#### 3.3.1. Chl a, Total Carot, and Polyphenol Quantification in Phospholipid Vesicles

Total amount of Chl a and carotenoids were assessed according to Milia et al. [62]. In brief, the particles were disrupted in methanol (1:50, *v/v*) and the supernatant after centrifugation was analyzed by a UV-Vis spectrophotometer (Cary 50, Varian Inc., Palo Alto, CA, USA) at 470, 665, and 750 nm. Total polyphenolic content was expressed as mg·g^−1^ Gallic ac. Eq (DW) and was determined by the Folin–Ciocalteu reagent method on 100 µL of methanolic extract and through UV–Vis spectrometer analysis at 765 nm (Cary 50, Varian Inc., Palo Alto, CA, USA) according to Manconi et al.’s methodology [63].

#### 3.3.2. Antioxidant Activity

The antioxidant activity was assessed by the 2, 2 diphenyl-1-picrylhydrazyl (DPPH) test according to Manca et al.’s methodology [64]. After dilution (1/50, *v/v*), 100 µL of methanolic extract was mixed with 900 µL of DPPH (25 μM in MeOH) and incubated in the dark at room temperature for 30 min, and the absorbance was measured at 517 nm using a UV–Vis spectrometer (Cary 50, Varian Inc., Milan, Italy). The test was carried out in triplicate and the antioxidant activity was expressed as a percentage with the following formula:

Antioxidant activity % = (ABS_DPPH_ − ABS_sample_)/ABS_DPPH_ ∗ 100 (1)

### 3.4. In Vitro Release of Spirulina Extract

Spirulina extract (2 mL) in aqueous dispersion or incorporated in Lps, Bls, or G-Bls was placed in polycarbonate dialysis tubes (Spectra/Por^®^ dialysis tubes: 12–14 kDa MW cut-off, with pores of 3 nm; Spectrum Laboratories Inc., Rancho Dominguez, CA, USA) and left under constant stirring (300 rpm) in a falcon filled with 25 mL of the acidic medium simulating pH gastric fluid (pH 1.2) at 37 °C for 2 h, with 25 mL of the neutral medium simulating pH intestinal fluid (pH 7.0) for 6 h. At selected times (0, 15, 45, 90, and 120 min in the gastric medium and 0, 1.5, 3, 4.5, and 6 h in the intestinal medium), a 20 µL aliquot was withdrawn for analysis. The released extract was quantified using the DPPH colorimetric method and expressed as a percentage of antioxidant activity over time. To maintain sink conditions, an equal volume of fresh medium was added after each sampling [65]. All experiments were performed in triplicate.

### 3.5. Cell Culture and Biocompatibility of Phospholipid Vesicles

The MTT colorimetric test was utilized to evaluate the biocompatibility of the dispersion and phospholipid vesicles [65]. The human colon adenocarcinoma (Caco-2, ATCC HTB-37, ATCC, Manassas, VA, USA) cells were cultured as monolayers in 75 cm^2^ flasks at 37 °C with 100% humidity and 5% carbon dioxide. The growth media used was Dulbecco’s Modified Eagle Medium (DMEM) containing glucose and L-glutamine. The media was supplemented with 10% fetal bovine serum, 1% penicillin (10,000 units·mL^−1^), and streptomycin (10,000 units·mL^−1^). Cells were cultured for 10 days to reach a fully differentiated state. Subsequently, cell division was halted for an additional 20 days. This step helped to maintain the differentiated state and prevented the cells from proliferating, which could alter their mature characteristics. Following these steps, the cells were treated with trypsin and then re-plated into 96-well plates at a density of 1 × 10^4^ cells per well and allowed to attach and stabilize for 24 h [64]. The cells were then exposed to Spirulina extract dispersion, Lip, Bls, and G-Bls at concentrations of 2, 20, and 200 μg·mL^−1^ for 48 h.

After the treatment period, the cell culture medium was removed and replaced with a solution of MTT (0.5 mg·mL^−1^ in PBS, pH 7.40). The MTT reagent was metabolized by viable cells to form insoluble formazan crystals. After three hours, the formazan crystals were dissolved in 100 μL of DMSO. The optical density of the resulting solution was measured at 570 nm using a microplate reader (Multiskan EX, Thermo Fisher Scientific, Inc., Waltham, MA, USA). Each sample was tested in triplicate, and untreated cells served as a positive control.

### 3.6. Protective Effect of Phospholipid Vesicles Against Damage Induced in Cells by Hydrogen Peroxide (H_2_O_2_)

The ability to protect adenocarcinoma cells (Caco-2, ATCC HTB-37, USA) from oxidative stress by hydrogen peroxide (H_2_O_2_) was determined according to Castangia et al. [53]. Caco-2 cells were seeded in 96-well plates at a density of 1 × 10^4^ cells per well and allowed to adhere for 24 h. The cells were added with Spirulina extract in dispersion or incorporated in Lps, Bls, and G-Bls at 20 and 200 μg mL^−1^ of extract and exposed to oxidative stress by adding H_2_O_2_ at 1 × 10^−3^%. Caco-2 cells treated with H_2_O_2,_ and untreated cells (representing 100% viability) were used as control samples. After 4 h of incubation, the cells were washed with PBS, and their viability was assessed using the MTT assay.

### 3.7. Statistical Analysis

All experiments were conducted in triplicate, and differences among the samples were evaluated using ANOVA, followed by Tukey’s post hoc test, to identify significant pairwise differences or Dunnett’s multiple comparisons (*p* < 0.05). Statistical analysis was performed with GraphPad Prims 8.3.0 (538).

## 4. Conclusions

Phospholipid vesicle systems incorporating Spirulina extract have been proved to exhibit optimal physicochemical attributes, characterized by uniform particle size, a low polydispersity index, a strong negative zeta potential, and high entrapment efficiency. These properties have been shown to remain constant over a 90-day storage period, thereby confirming the long-term stability of the systems. All formulations afforded protection of the active ingredient under gastric acidity and enabled release at intestinal pH, confirming their suitability for oral delivery. Notably, G-Bls exhibited superior biocompatibility on Caco-2 monolayers, an effect attributed to gelatin-mediated mucoadhesive interactions that enhanced cellular uptake while mitigating the cytotoxicity typically associated with lipid carriers. Antioxidant evaluations revealed that vesicular preparations supported radical-scavenging efficacy comparable to the free extract, showing the effective release and preservation of bioactivity. Collectively, these results showed G-Bls as a delivery platform with considerable potential for the delivery of Spirulina-based nutraceuticals. It is recommended that future investigations include in vivo studies with the aim of elucidating bioavailability, pharmacokinetics, and therapeutic outcomes. Such studies should also assess interactions with the gut microbiota, optimize dosing regimens, and address long-term safety. Furthermore, it is essential to address commercial translation challenges, specifically process scalability, real-world stability, and regulatory compliance.

## Figures and Tables

**Table 1:** Characterization of vesicles: mean diameter (MD), polydispersity index (PDI), zeta potential (ZP), and entrapment efficiency (EE%).

Vesicle	MD (nm)	PDI	ζ Potential (mV)	EE (%) DPPH	EE (%) Tot. Polyphen	EE (%) Tot. Carot	EE (%) Chl a
Lps	101.8 ± 0.5 a *	0.2 a	−31.9 ± 5.2 a	94.7 ± 6.0 a	94.4 ± 11.2 a	99.2 ± 11.5 a	84.2 ±8.3 a
Bls	103.2 ± 1.5 a	0.2 a	−31.1 ± 6.4 a	89.5 ± 5.3 a	94.3 ± 10.3 a	89.9 ± 4.6 a	85.2 ± 11.0 a
G-Bls	129.7 ± 1.2 b (e)	0.3 b (c)	−31.3 ± 4.2 a	95.4 ± 3.8 a	82.1 ± 2.7 a	91.1 ± 1.8 a	86.6 ± 6.0 a

Mean values belong to 6 independent replicates. * Different letters in the column indicate statistical differences. Letter in brackets identified as *p* values (c: *p* < 0.01; e: *p* < 0.0001).

**Table 2:** The characterization of freeze-dried vesicles with mannitol added at 120 and 220 mg·mL^−1^ by means of mean diameter (MD), the polydispersity index (PDI), and the zeta potential (ZP).

Phospholipid Vesicles	Mannitol (mg·mL^−1^)	Days	MD (nm)	PDI	ζ Potential (mV)
Lps	120	0	186.2 a *	0.29 a	−31.7 a
		90	199.8 a (a)	0.29 a (ab)	−29.3 a (ab)
	220	0	147.8 a	0.31 a	−31.3 a
		90	154.7 a (b)	0.32 a (a)	−31.3 a (a)
Bls	120	0	137.1 a	0.18 a	−31.7 a
		90	137.1 a (c)	0.18 a (c)	−31.7 a (a)
	220	0	115.0 a	0.17 a	−31.3 a
		90	121.0 a	0.22 a (c)	−28.7 a (b)
G-Bls	120	0	134.7 a	0.29 a	−30.7 a
		90	139.5 a (c)	0.31 a (a)	−31.3 a (a)
	220	0	131.8 a	0.22 a	−28.7 a
		90	131.6 a (c)	0.22 a (bc)	−30.3 a (ab)

Mean values ± standard deviations from at least 6 replicates are reported. * Different letters indicate statistical differences within vesicles among days of storage (T = 0, T = 90); letters in brackets indicate statistical differences among vesicles with equal mannitol addition at 90 d (*p* > 0.05). No significant differences were observed (*p* > 0.05) between day 0 and day 90 for the same formulation at the same mannitol concentration.

**Table 3:** The physicochemical properties of vesicles, measured after exposure for 2 h at pH 1.2 and 6 h at pH 7.0.

Vesicles	pH	Time	MD (nm)	PDI
Lps	Freshly prepared	-	101.8 ± 0.5 c	0.19 b
	1.2	2 h	141.0 ± 1.9 b **	0.32 a *
	7.0	6 h	163.4 ± 1.6 ab **	0.39 a *
Bls	Freshly prepared	-	103.2 ± 1.6 c	0.17 c
	1.2	2 h	142.9 ± 3.2 b ****	0.30 b *
	7.0	6 h	115.3 ± 1.2 a **	0.47 a ***
G-Bls	Freshly prepared	-	129.7 ± 1.2 b	0.33 a
	1.2	2 h	239.7 ± 2.9 a ****	0.22 b **
	7.0	6 h	221.2 ± 1.3 a ****	0.20 b ***

Different letters indicate statistical differences within vesicles among trials (pH 1.2, and pH 7.0) and with freshly prepared samples (* *p* < 0.05; ** *p* < 0.01; *** *p* < 0.001; **** *p* < 0.0001).

**Table 4:** The compositions of Lps, Bls, and G-Bls vesicles.

	Extract	S75	Cholesterol	SDC	Gelatine	PBS
	mg·mL^−1^					mL
Lps	20	120	20	-	-	2
Bls	20	120	20	20	-	2
G-Bls	20	120	20	20	80	2

## Data Availability

The original contributions presented in the study are included in the article. Further inquiries can be directed to the corresponding authors.

## References

[1] Najar-Almanzor C.E., Velasco-Iglesias K.D., Nunez-Ramos R., Uribe-Velázquez T., Solis-Bañuelos M., Fuentes-Carrasco O.J., Chairez I., García-Cayuela T., Carrillo-Nieves D. (2023). Microalgae-assisted green bioremediation of food-processing wastewater: A sustainable approach toward a circular economy concept. J. Environ. Manag..

[2] Lafarga T., Sánchez-Zurano A., Villaró S., Morillas-España A., Acién G. (2021). Industrial production of Spirulina as a protein source for bioactive peptide generation. Trends Food Sci. Technol..

[3] Shah M.A.R., Zhu F., Cui Y., Hu X., Chen H., Kayani S.I., Huo S. (2024). Mechanistic insights into the nutritional and therapeutic potential of *Spirulina* (*Arthrospira* spp.): Challenges and opportunities. Trends Food Sci. Technol..

[4] Spínola M.P., Mendes A.R., Prates J.A. (2024). Chemical composition, bioactivities, and applications of *Spirulina* (*Limnospira platensis*) in food, feed, and medicine. Foods.

[5] Anvar A.A., Nowruzi B. (2021). Bioactive properties of Spirulina: A review. Microb. Bioact..

[6] Bortolini D.G., Maciel G.M., Fernandes I.D.A.A., Pedro A.C., Rubio F.T.V., Branco I.G., Haminiuk C.W.I. (2022). Functional properties of bioactive compounds from Spirulina spp.: Current status and future trends. Food Chem. Mol. Sci..

[7] Calella P., Cerullo G., Di Dio M., Liguori F., Di Onofrio V., Galle F., Liguori G. (2022). Antioxidant, anti-inflammatory, and immunomodulatory effects of *Spirulina* in exercise and sport: A systematic review. Front. Nutr..

[8] Supriya R., Delfan M., Saeidi A., Samaie S.S., Al Kiyumi M.H., Escobar K.A., Zouhal H. (2023). Spirulina supplementation with high-intensity interval training decreases adipokine levels and cardiovascular risk factors in men with obesity. Nutrients.

[9] Krishnan H., Kaushik D., Kumar M., Öz E., Brennan C., Proestos C., Kumar V., Ahmed M., Özç F. (2024). Exploring the natural efficacy of spirulina powder for combating obesity, diabetes, and inflammation. J. Sci. Food Agric..

[10] Gentscheva G., Nikolova K., Panayotova V., Peycheva K., Makedonski L., Slavov P., Yotkovska I. (2023). Application of Arthrospira platensis for medicinal purposes and the food industry: A review of the literature. Life.

[11] Chaouachi M., Vincent S., Groussard C. (2024). A review of the health-promoting properties of Spirulina with a focus on athletes’ performance and recovery. J. Diet. Suppl..

[12] Kaufman M.W., Roche M., Fredericson M. (2022). The impact of supplements on sports performance for the trained athlete: A critical analysis. Curr. Sports Med. Rep..

[13] McBurney M.I., Blumberg J.B., Costello R.B., Eggersdorfer M., Erdman J.W., Harris W.S., Schurgers L.J. (2021). Beyond nutrient deficiency—Opportunities to improve nutritional status and promote health modernizing DRIs and supplementation recommendations. Nutrients.

[14] Wegierska A.E., Charitos I.A., Topi S., Potenza M.A., Montagnani M., Santacroce L. (2022). The connection between physical exercise and gut microbiota: Implications for competitive sports athletes. Sports Med..

[15] Cataldi S., Bonavolontà V., Poli L., Clemente F.M., De Candia M., Carvutto R., Fischetti F. (2022). The relationship between physical activity, physical exercise, and human gut microbiota in healthy and unhealthy subjects: A systematic review. Biology.

[16] Guan F., Fu G., Ma Y., Zhou L., Li G., Sun C., Zhang T. (2024). Spirulina polysaccharide-based prebiotic foods preparations—A promising approach for modulating gut microbiota and improving health. J. Funct. Foods.

[17] Trotta T., Porro C., Cianciulli A., Panaro M.A. (2022). Beneficial effects of Spirulina consumption on brain health. Nutrients.

[18] Abd Elkader H.T.A.E., Essawy A.E., Al-Shami A.S. (2024). Bioactive compounds of the genus Spirulina can prevent the progression of neurological diseases. Neurochem. J..

[19] Fernandes F.A., Carocho M., Prieto M.A., Barros L., Ferreira I.C.F.R., Heleno S.A. (2024). Nutraceuticals and dietary supplements: Balancing out the pros and cons. Food Funct..

[20] Aquino R.P., Auriemma G., Conte G.M., Esposito T., Sommella E., Campiglia P., Sansone F. (2020). Development of chitosan/mannitol microparticles as a delivery system for the oral administration of a Spirulina bioactive peptide extract. Molecules.

[21] Adjali A., Clarot I., Chen Z., Marchioni E., Boudier A. (2022). Physicochemical degradation of phycocyanin and means to improve its stability: A short review. J. Pharm. Anal..

[22] Liu L., McClements D.J., Liu X., Liu F. (2024). Overcoming biopotency barriers: Advanced oral delivery strategies for enhancing the efficacy of bioactive food ingredients. Adv. Sci..

[23] Zhang F., Li Z., Duan Y., Abbas A., Mundaca-Uribe R., Yin L., Wang J. (2022). Gastrointestinal tract drug delivery using algae motors embedded in a degradable capsule. Sci. Robot..

[24] European Parliament and Council (2002). Regulation (EC) No 178/2002 of the European Parliament and of the Council of 28 January 2002. Official Journal of the European Communities, L 31, 1–24. https://eur-lex.europa.eu/legal-content/EN/TXT/?uri=CELEX%3A32002R0178.

[25] Coppens P., Biesalski H.K., Koletzko B. (2020). The importance of food supplements for public health and well-being. Hidden Hunger and the Transformation of Food Systems: How to Combat the Double Burden of Malnutrition?.

[26] 2024 CRN Consumer Survey on Dietary Supplements. https://www.nutritionaloutlook.com/view/council-for-responsible-nutrition-discloses-2024-consumer-survey-results-showcasing-increasing-of-specialty-product-usage.

[27] Djaoudene O., Romano A., Bradai Y.D., Zebiri F., Ouchene A., Yousfi Y., Madani K. (2023). A global overview of dietary supplements: Regulation, market trends, usage during the COVID-19 pandemic, and health effects. Nutrients.

[28] Barani M., Sangiovanni E., Angarano M., Rajizadeh M.A., Mehrabani M., Piazza S., Gangadharappa H.V., Pardakhty A., Mehrbani M., Dell’Agli M. (2021). Phytosomes as Innovative Delivery Systems for Phytochemicals: A Comprehensive Review of Literature. Intern. J. Nanomed..

[29] Šturm L., Poklar Ulrih N. (2021). Basic Methods for Preparation of Liposomes and Studying Their Interactions with Different Compounds, with the Emphasis on Polyphenols. Int. J. Mol. Sci..

[30] He H., Lu Y., Qi J., Zhu Q., Chen Z., Wu W. (2019). Adapting liposomes for oral drug delivery. Acta Pharm. Sin. B.

[31] Mondal D., Mandal R.P., De S. (2022). Addressing the superior drug delivery performance of bilosomes—A microscopy and fluorescence study. ACS Appl. Bio Mater..

[32] Nayak D., Rathnanand M., Tippavajhala V.K. (2023). Unlocking the potential of bilosomes and modified bilosomes: A comprehensive journey into advanced drug delivery trends. AAPS PharmSciTech.

[33] Zhang B., Xue A., Zhang C., Yu J., Chen W., Sun D. (2016). Bile salt liposomes for enhanced lymphatic transport and oral bioavailability of paclitaxel. Die Pharm..

[34] Jain S., Harde H., Indulkar A., Agrawal A.K. (2014). Improved stability and immunological potential of tetanus toxoid containing surface engineered bilosomes following oral administration. Nanomedicine.

[35] Milano F., Masi A., Madaghiele M., Sannino A., Salvatore L., Gallo N. (2023). Current trends in gelatin-based drug delivery systems. Pharmaceutics.

[36] Parashar P., Kumar P., Gautam A.K., Singh N., Bera H., Sarkar S., Saraf S.A., Saha S., Grumezescu A.M. (2021). Gelatin-based nanomaterials in drug delivery and biomedical applications. Biopolymer-Based Nanomaterials in Drug Delivery and Biomedical Applications.

[37] Liang C., Du J., Hou T., Sui L., Li J., Zhao Y., Wu D. (2024). Improvement of membrane stabilizer on the rehydrated reconstruction of spray-dried mannitol-based liposome powder. Colloid Polym. Sci..

[38] Gupta D.K., Ahad A., Waheed A., Aqil M., Al-Jenoobi F.I., Al-Mohizea A.M., Iqbal Z., Baboota S. (2022). Bilosomes: A Novel Platform for Drug Delivery. Systems of Nanovesicular Drug Delivery.

[39] Singh S., Dash A.K., Singh S., Dash A.K. (2024). Physical properties, their determination, and importance in pharmaceutics. Pharmaceutics, Basic Principles and Application to Pharmacy Practice.

[40] Danaei M., Dehghankhold M., Ataei S., Hasanzadeh Davarani F., Javanmard R., Dokhani A., Mozafari M.R. (2018). Impact of particle size and polydispersity index on the clinical applications of lipidic nanocarrier systems. Pharmaceutics.

[41] Lowry G.V., Hill R.J., Harper S., Rawle A.F., Hendren C.O., Klaessig F., Rumble J. (2016). Guidance to improve the scientific value of zeta-potential measurements in nano EHS. Environ. Sci. Nano.

[42] Nsairat H., Khater D., Sayed U., Odeh F., Al Bawab A., Alshaer W. (2022). Liposomes: Structure, composition, types, and clinical applications. Heliyon.

[43] Ansari I., Shakeel K. (2023). A review on bilosomes: Advanced drug delivery system. Int. J. Pharm. Sci. Med..

[44] Machado A.R., Pinheiro A.C., Vicente A.A., Souza-Soares L.A., Cerqueira M.A. (2019). Liposomes loaded with phenolic extracts of Spirulina LEB-18: Physicochemical characterization and behaviour under simulated gastrointestinal conditions. Food Res. Int..

[45] Zewail M., Gaafar P.M., Youssef N.A.H.A., Ali M.E., Ragab M.F., Kamal M.F., Abbas H. (2022). Novel *Spirulina platensis* bilosomes for combating UVB-induced skin damage. Pharmaceuticals.

[46] Allaw M., Perra M., Parekh P., Serra M., Marongiu J., Castangia I., Fulgheri F., Caboni P., Tolle G., Corrias F. (2025). Antioxidant and neuroprotective effects of nutriosomes and grape pomace phytochemicals in a cell model of oxidative stress and mouse model of Parkinson disease. Sci. Rep..

[47] Păvăloiu R.D., Sha’at F., Neagu G., Deaconu M., Bubueanu C., Albulescu A., Sha’at M., Hlevca C. (2021). Encapsulation of Polyphenols from *Lycium barbarum* Leaves into Liposomes as a Strategy to Improve Their Delivery. Nanomaterials.

[48] Agarry I.E., Wang Z., Cai T., Wu Z., Kan J., Chen K. (2022). Utilization of different carrier agents for chlorophyll encapsulation: Characterization and kinetic stability study. Food Res. Int..

[49] Hamadou A.H., Huang W.C., Xue C., Mao X. (2020). Comparison of β-Carotene Loaded Marine and Egg Phospholipids Nanoliposomes. J. Food Eng..

[50] Sonje J., Thakral S., Mayhugh B., Sacha G., Nail S., Srinivasan J., Suryanarayanan R. (2022). Mannitol Hemihydrate in Lyophilized Protein Formulations: Impact of Its Dehydration during Storage on Sucrose Crystallinity and Protein Stability. Int. J. Pharm..

[51] Zhu S., Wang X., Jin Y., Peng N., Wei Z., Lian J., Zhou X. (2025). Dual Cryoprotection of Gelatin–Tea Polyphenol Microgels on Surimi by Targeting Ice Inhibition and Component Stabilization. Food Chem..

[52] Trenkenschuh E., Friess W. (2021). Freeze-Thaw Stability of Aluminium Oxide Nanoparticles. Int. J. Pharm..

[53] Castangia I., Corrias F., Jiménez F.J.L., Aroffu M., Fulgheri F., Perra M., Manca M.L. (2024). Formulation and testing of cutting-edge food supplements tailored for glucose and oxidation controlling, converting artichoke by-products in inulin-rich antioxidant phytocomplex loaded into zein liposomes. Food Biosci..

[54] Muhaimin M., Chaerunisaa A.Y., Bodmeier R. (2022). Impact of dispersion time interval and particle size on release profiles of propranolol HCl and carbamazepines from microparticle blends system. Sci Rep..

[55] Battogtokh G., Joo Y., Abuzar S.M., Park H., Hwang S.J. (2022). Gelatin coating for the improvement of stability and cell uptake of hydrophobic drug-containing liposomes. Molecules.

[56] Jia X., Fan X., Chen C., Lu Q., Zhou H., Zhao Y., Geng H. (2024). Chemical and Structural Engineering of Gelatin-Based Delivery Systems for Therapeutic Applications: A Review. Biomacromolecules.

[57] (2009). Biological Evaluation of Medical Devices—Part 5: Tests for In Vitro Cytotoxicity.

[58] United States Pharmacopeial Convention (2006). USP 24–NF 19. United States Pharmacopeia–National Formulary.

[59] Milia M., Pasquini V., Addis P., Angioni A. (2025). Eco-Friendly Extraction to Enhance Antioxidants and Nutritional Value in *Arthrospira platensis*. Foods.

[60] Castangia I., Manca M.L., Caddeo C., Maxia A., Murgia S., Pons R., Manconi M. (2015). Faceted Phospholipid Vesicles Tailored for the Delivery of Santolina insularis Essential Oil to the Skin. Colloids Surf. B Biointerfaces.

[61] Paseban K., Noroozi S., Gharehcheloo R., Haddadian A., Robattorki F.F., Dibah H., Gharaghie T.P. (2024). Preparation and optimization of niosome encapsulated meropenem for significant antibacterial and anti-biofilm activity against methicillin-resistant Staphylococcus aureus isolates. Heliyon.

[62] Milia M., Corrias F., Addis P., Chini Zitelli G., Cicchi B., Torzillo G., Andreotti V., Angioni A. (2022). Influence of Different Light Sources on the Biochemical Composition of Arthrospira spp. Grown in Model Systems. Foods.

[63] Manconi M., Marongiu F., Castangia I., Manca M.L., Caddeo C., Tuberoso C.I.G., Fadda A.M. (2016). Polymer-associated liposomes for the oral delivery of grape pomace extract. Colloids Surf. B Biointerfaces.

[64] Manca M.L., Castangia I., Zaru M., Nácher A., Valenti D., Fernàndez-Busquets X., Manconi M. (2015). Development of curcumin loaded sodium hyaluronate immobilized vesicles (hyalurosomes) and their potential on skin inflammation and wound restoring. Biomaterials.

[65] Manca M.L., Zaru M., Manconi M., Lai F., Valenti D., Sinico C., Fadda A.M. (2013). Glycerosomes: A new tool for effective dermal and transdermal drug delivery. Int. J. Pharma..

